# Morphine Suppresses IFN Signaling Pathway and Enhances AIDS Virus Infection

**DOI:** 10.1371/journal.pone.0031167

**Published:** 2012-02-16

**Authors:** Yizhong Wang, Xu Wang, Li Ye, Jieliang Li, Li Song, Nilija Fulambarkar, Wenzhe Ho

**Affiliations:** 1 Department of Pathology and Laboratory Medicine, Temple University School of Medicine, Philadelphia, Pennsylvania, United States of America; 2 The Center for Animal Experiment/ABSL-3 Laboratory, Wuhan University, Hubei, People's Republic of China; University of Nebraska Medical Center, United States of America

## Abstract

**Background:**

Opioids exert a profound influence on immunomodulation and enhance HIV infection and replication. However, the mechanism(s) of their action remains to be determined. We thus investigated the impact of morphine on the intracellular innate antiviral immunity.

**Methodology/Principal Findings:**

Seven-day-cultured macrophages were infected with equal amounts of cell-free HIV Bal or SIV Delta_B670_ for 2 h at 37°C after 24 h of treatment with or without morphine. Effect of morphine on HIV/SIV infection and replication was evaluated by HIV/SIV RT activity assay and indirect immunofluorescence for HIV p24 or SIV p28 antigen. The mRNA expression of cellular factors suppressed or induced by morphine treatment was analyzed by the real-time RT-PCR. We demonstrated that morphine treatment of human blood monocyte-derived macrophages significantly inhibited the expression of interferons (IFN-α, IFN-β and IFN-λ) and IFN-inducible genes (APOBEC3C/3F/3G and 3H). The further experiments showed that morphine suppressed the expression of several key elements (RIG-I and IRF-7) in IFN signaling pathway. In addition, morphine treatment induced the expression of suppressor of cytokine signaling protein-1, 2, 3 (SOCS-1, 2, 3) and protein inhibitors of activated STAT-1, 3, X, Y (PIAS-1, 3, X, Y), the key negative regulators of IFN signaling pathway.

**Conclusions:**

These findings indicate that morphine impairs intracellular innate antiviral mechanism(s) in macrophages, contributing to cell susceptibility to AIDS virus infection.

## Introduction

Injection drug users (IDUs) are at a significant high risk for aquiring HIV infection and contribute to the spread of the virus [1], [2]. Several early studies indicated that intravenous use of opiates influences the outcome of HIV infection [1], [2], [3], [4], [5]. IDUs frequently involve the abuse of heroin, the most common abused opiate. Heroin injection increased the risk of acquiring HIV [6] and progression to AIDS [3]. However, because of the extreme complexity of opiate addition and/or HIV infection, it has been extremely difficult to compare different clinical and epidemiological findings in studying the impact of opiate abuse on HIV disease progression [7]. In contrast, laboratory *in vitro* studies have yielded relatively agreeable data, showing that morphine, the active metabolite of heroin, enhances susceptibility of the immune cells to HIV infection. Peterson et al. first reported that morphine enhances HIV replication in human PBMC coculture system [8]. Several studies [9], [10], [11], [12], [13], [14], [15] showed that morphine could activate mu opioid receptors of human immune cells (macrophages, T lymphocytes, microglia) and up-regulate the expression of CCR5 and CXCR4, the key HIV entry coreceptors. Morphine-mediated induction of CCR5 and CXCR4 was associated with increased HIV infection of macrophages [10], [16]. Morphine also enhanced simian immunodeficiency virus (SIV) infection and replication in both *in vivo* and *in vitro* systems. Morphine treatment increased SIV replication in CEM×174 cells [17]. Injection with morphine enhanced SIV replication in *Rhesus Macaques* [18]. Induction of CCR5 expression in monkey peripheral mononuclear cells by morphine contributes to enhanced SIV replication [14]. However, it has also been reported that morphine treatment slowed SIV disease progression [7], [19], [20].

Although the role of opiates in promoting HIV disease progression is still debatable, overwhelming evidence indicates that heroin and other opiate derived substances affect both adaptive and innate immunity [20], [21], [22], [23]. Innate immunity is the first line of the defense mechanism against viral infections. Interferons (IFNs) are key players in host innate immunity, as they possess antiviral activity against a variety of viruses [24], including HIV [25], [26]. While both type I IFNs (IFN-α, -β, -ω, -κ, -ε, -τ, -δ and -ν subtypes) and type II IFN (IFN-γ) have been known for decades as the antiviral cytokines, a novel class of cytokines (interleukin-28/29) was recently discovered and named as type III IFNs (also called IFN-λ) [27]. Although IFN-λ exerts its action through a receptor complex distinct from that for the type I IFNs [28], [29], [30], [31], IFN-λ shares a number of common biological functions with type I IFNs. Similar to type I IFNs, IFN-λ has potent antiviral activity against viral infections [32], [33], [34], including HIV [26]. Given the critical role of IFNs control of HIV replication, it is of importance to determine the specific impact of opiates on IFN signaling pathway and the mechanisms responsible for the actions.

## Materials and Methods

### Cell culture

Peripheral blood was purchased from the Center for AIDS Research at the University of Pennsylvania. The protocol used to isolate blood from donors, purify the blood components, and distribute this material to the investigators was approved by the IRB of the Center for AIDS Research. These blood samples were screened for all normal blood-borne pathogens and certified to be pathogen free. Monocytes were purified from peripheral blood of three healthy adult donors according to our previously described technique [35]. Freshly isolated monocytes were cultured in 48-well culture plates at a density of 2.5×10^5^ cells/well in Dulbecco modified Eagle medium (DMEM) containing 10% fetal calf serum. Macrophages refer to 7-day-cultured monocytes *in vitro*.

### Reagents

Morphine sulfate was obtained from National Institute on Drug Abuse (Rockville, MD). Naltrexone was obtained from Sigma (St Louis, MO). Mouse anti-HIV p24 monoclonal obtained from the AIDS Research and Reference Reagent Program (NIH, Bethesda, MD). Mouse anti-SIV p28 monoclonal antibody was purchased from Fitzgerald Industries (Acton, MA). Alexa Fluor 488 goat anti-mouse IgG and Hoechst 33342 were purchased from Invitrogen (Carlsbad, CA).

### Morphine and/or naltrexone treatment

Seven-day-cultured macrophages (2.5×10^5^cells/well) were treated with or without morphine at different concentrations (10^−12^ to 10^−8^ M) for different time points (3–24 h). To investigate whether naltrexone antagonizes the morphine action, we used naltrexone (10^−8^ M) to treat macrophages for 1 h followed by morphine treatment. There were no cytotoxic effects of morphine and naltrexone treatment on macrophages as demonstrated by trypan blue dye staining (data not shown).

### Infection of macrophages with HIV Bal strain or SIV Delta_B670_ strain

HIV Bal strain and SIV Delta_B670_ strain were obtained from the AIDS Research and Reference Reagent Program (NIH, Bethesda, MD). Macrophages were infected with equal amounts of cell-free HIV Bal (p24 20 ng/10^6^ cells) or SIV Delta_B670_ (p28 20 ng/10^6^ cells) for 2 h at 37°C after 24 h of treatment with or without morphine. The cells were then washed three times with Dulbecco's modified Eagle's medium to remove unabsorbed virus, and fresh media containing morphine and/or naltrexone were added to the cell cultures. The final wash was tested for HIV/SIV reverse transcriptase (RT) activity and shown to be free of residual inocula. Untreated cells served as a control. Culture supernatants were collected for HIV/SIV RT activity assay at days 3, 6, 9, 12 and 15 after infection.

### HIV/SIV RT assay

HIV and SIV RT activity was determined based on the technique of [36] with modifications [37]. In brief, 10 µl of culture supernatants from macrophages infected with or without HIV/SIV was added to a cocktail containing poly(A), oligo(dT) (Amersham Biosciences, Inc., Piscataway, NJ), MgCl_2_, and [32P]dTTP (Amersham Biosciences, Inc.) and incubated for 20 h at 37°C. Then, 30 µl of the cocktail was spotted onto DE81 paper (Whatman Internatianl Ltd, England), dried and washed five times with 2× saline-sodium citrate buffer and once with 95% ethanol. The filter paper was then air-dried. Radioactivity was counted in a liquid scintillation counter (PerkinElmer Life Sciences, Boston, MA).

### RNA extraction and real-time RT-PCR

Total RNA from macrophages was extracted with Tri-Reagent (Molecular Research Center, Cincinnati, OH) as previously described [38]. Total RNA (1 µg) was subjected to RT using the RT system (Promega, Madison, WI) with random primers for 1 h at 42°C. The reaction was terminated by incubating the reaction mixture at 99°C for 5 min, and the mixture was then kept at 4°C. The resulting cDNA was then used as a template for real-time PCR quantification. Real-time PCR was performed with 1/10 of the cDNA with the iQ SYBR Green Supermix (Bio-Rad Laboratories, Hercules, CA) as previously described [39]. The amplified products were visualized and analyzed using the software MyiQ provided with the thermocycler (iCycler iQ real time PCR detection system; Bio-Rad Laboratories). The oligonucleotide primers were synthesized by Integrated DNA Technologies, Inc. (Coralville, IA) and sequences will be available upon request. The cDNA was amplified by PCR and the products were measured using SYBR green I (Bio-Rad Laboratories, Inc., Hercules, CA). The data were normalized to glyceraldehyde-3-phosphate dehydrogenase (GAPDH) and presented as the change in induction relative to that of untreated control cells.

### Immunofluorescence assay

The macrophages infected with HIV Bal or SIV Delta_B670_ strain were cultured on glass coverslips at adensity of 2.5×10^5^/well in 48-well plates. The macrophages were washed with 1× cold PBS (with Ca^2+^ and Mg^2+^) twice. Cells were fixed at 4°C in 4% paraformaldehyde-4% sucrose in PBS for 20 min followed by 0.2% Triton X-100 for additional 10 min. Cells were blocked in Block Solution (Pierce, Rockford, IL) for 1 h at room temperature. To examine expression of HIV p24 or SIV p28, the fixed cells were stained with mouse anti-HIV p24 (1∶500) or mouse anti-SIV p28 monoclonal antibody (1∶500). After washing five times with 1× PBS, the cells were incubated with fluorescein isothiocyanate-conjugated goat anti-mouse IgG antibody (green, 1∶100) for 1 h. The cells were then mounted on a glass coverslip in mounting media (Biomeda, Foster City, CA) and viewed with a fluorescence microscopy (Zeiss, Jena, Germany). Hoechst 33342 was used for nuclei staining.

### Statistical analysis

Student's t-test was used to evaluate the significance of difference between groups, and multiple comparisons were performed by regression analysis and one-way analysis of variance. P values of less than 0.05 were considered significant. All data are presented as mean ± SD. Statistical analyses were performed with SPSS 11.5 for Windows. Statistical significance was defined as P<0.05.

## Results

### Morphine enhances AIDS virus infection of macrophages

**Figure 1:**
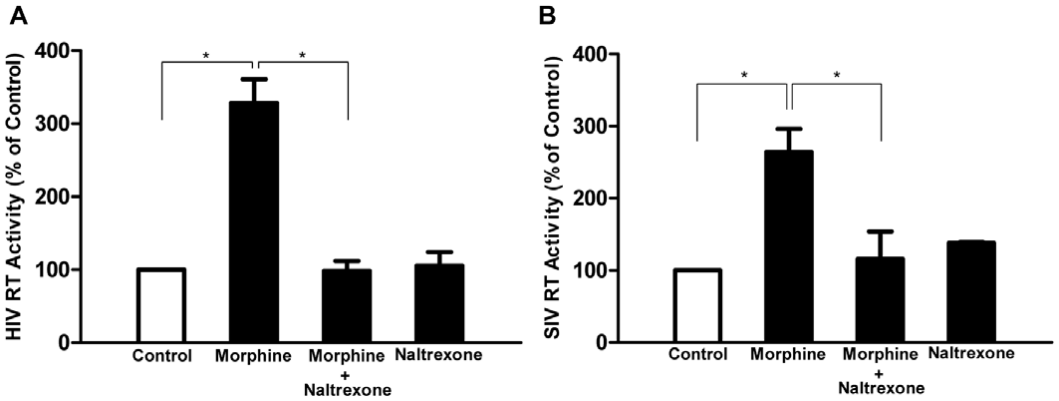
Morphine enhances HIV Bal strain (A) and SIV Delta_B670_ strain (B) infection of macrophages. Seven-day-cultured macrophages were incubated with or without morphine (10^−10^ M) for 24 h before HIV or SIV infection. An opioid receptor antagonist, naltrexone (10^−8^ M) was added to macrophage cultures 1 h before morphine (10^−10^ M) treatment. HIV or SIV RT activity in culture supernatant was determined at day 6 postinfection. Data are expressed as HIV (A) and SIV (B) RT activity in morphine-treated cells (percentage of control) to those in untreated cells, morphine-treated cells plus naltrexone versus morphine only. The results represent the mean ± SD of three experiments using cells from three different donors. Statistical analysis was performed using one-way analysis of variance, and significance is shown with * P<0.05 (morphine vs control or morphine vs morphine + naltrexone).

**Figure 2:**
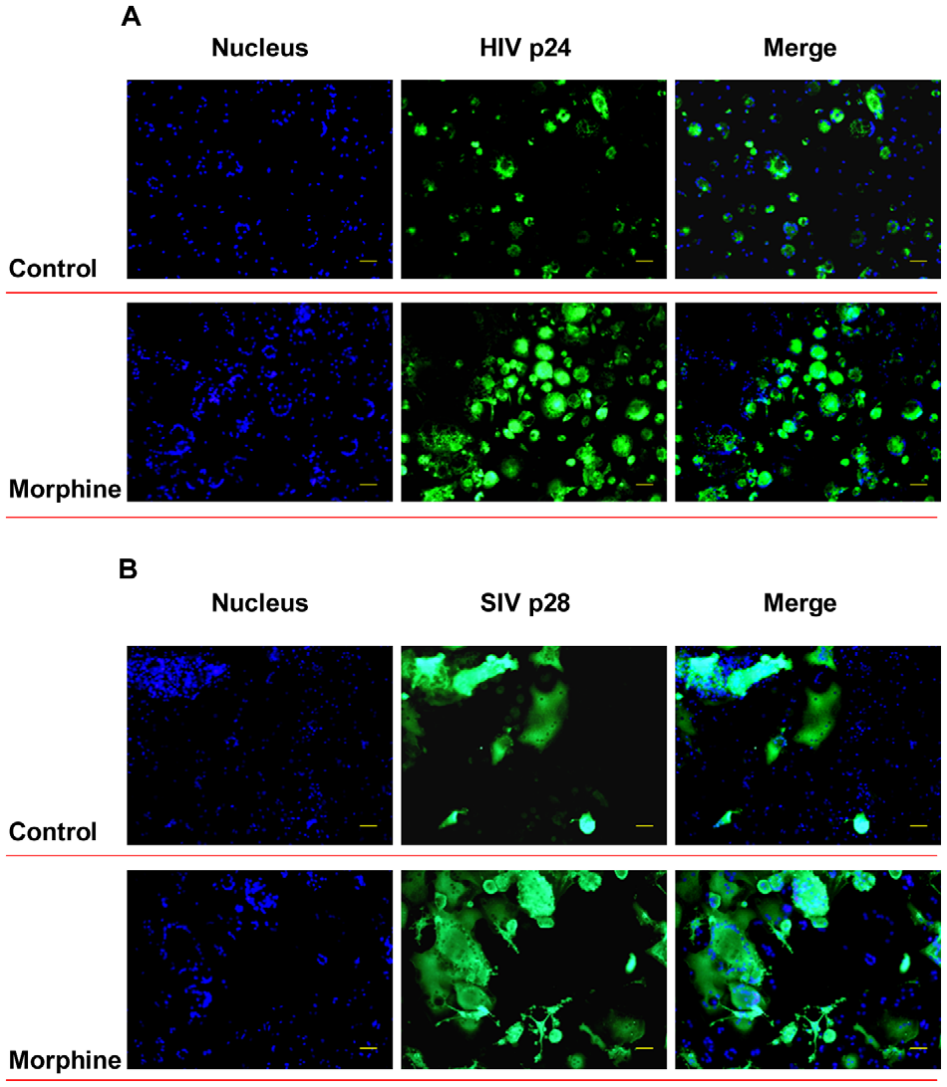
Effects of morphine on HIV p24 protein or SIV p28 protein expression in macrophages. Seven-day-cultured macrophages were treated with or without morphine (10^−10^ M) for 24 h and then incubated with HIV Bal strain or SIV Delta_B670_ strain for 2 h in the presence or absence of morphine (10^−10^ M). HIV p24 (A) or SIV p28 (B) protein expression in macrophages at day 15 postinfection was determined by immunofluoresence staining with antibodies against HIV p24 or SIV p28 protein (green). The nuclei were stained with Hoechst 33342 (blue) (magnification, 100×; scale bar: 100 µm).

**Figure 3:**
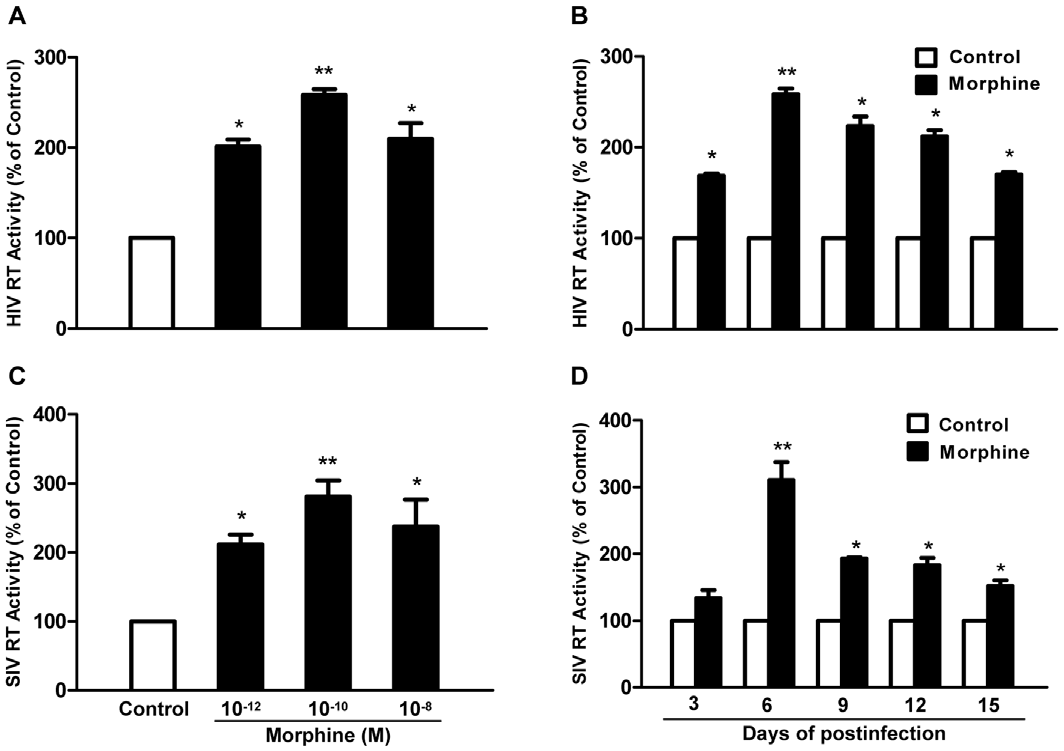
Dose-dependent and time-course effects of morphine on AIDS virus replication. A and C: Dose-dependent effect of morphine on HIV or SIV replication. Seven-day-cultured macrophages were treated with or without morphine at indicated concentrations for 24 h and then incubated with HIV Bal or SIV Delta_B670_ strain for 2 h in the presence or absence of morphine. Day 6 culture supernatant was collected for HIV (A) or SIV (C) RT assay. B and D: Time-course effect of morphine on HIV or SIV. Seven-day-cultured macrophages were treated with or without morphine (10^−10^ M) for 24 h prior to infection with HIV Bal strain or SIV Delta_B670_ strain for 2 h and then cultured for 15 days. HIV (B) or SIV (D) RT activity was determined in cultured supernatants at indicated time points postinfection. Data are expressed as HIV or SIV RT activity in morphine-treated cells (percentage of control) compared with those in untreated cells. The results represent the mean ± SD of three independent experiments using macrophages from three different donors. Statistical analysis was performed by one-way analysis of variance (A, C) or Student's t-test (B, D), and significance is shown morphine versus control with * (P<0.05) and ** (P<0.01).

We first determined the effect of morphine on AIDS virus (HIV and SIV) infection of macrophages. The addition of morphine to the cultures resulted in an increase in HIV RT activity (Fig. 1A) and viral protein expression (Fig. 2A). Similarly, morphine treatment enhanced SIV Delta_B670_ replication (Fig. 1B) and viral protein expression in macrophages (Fig. 2B). These effects of morphine on HIV or SIV were time- and dose-dependent (Fig. 3) and could be abrogated by naltrexone (Fig. 1A and 1B).

### Morphine suppresses intracellular type I and type III IFN expression

**Figure 4:**
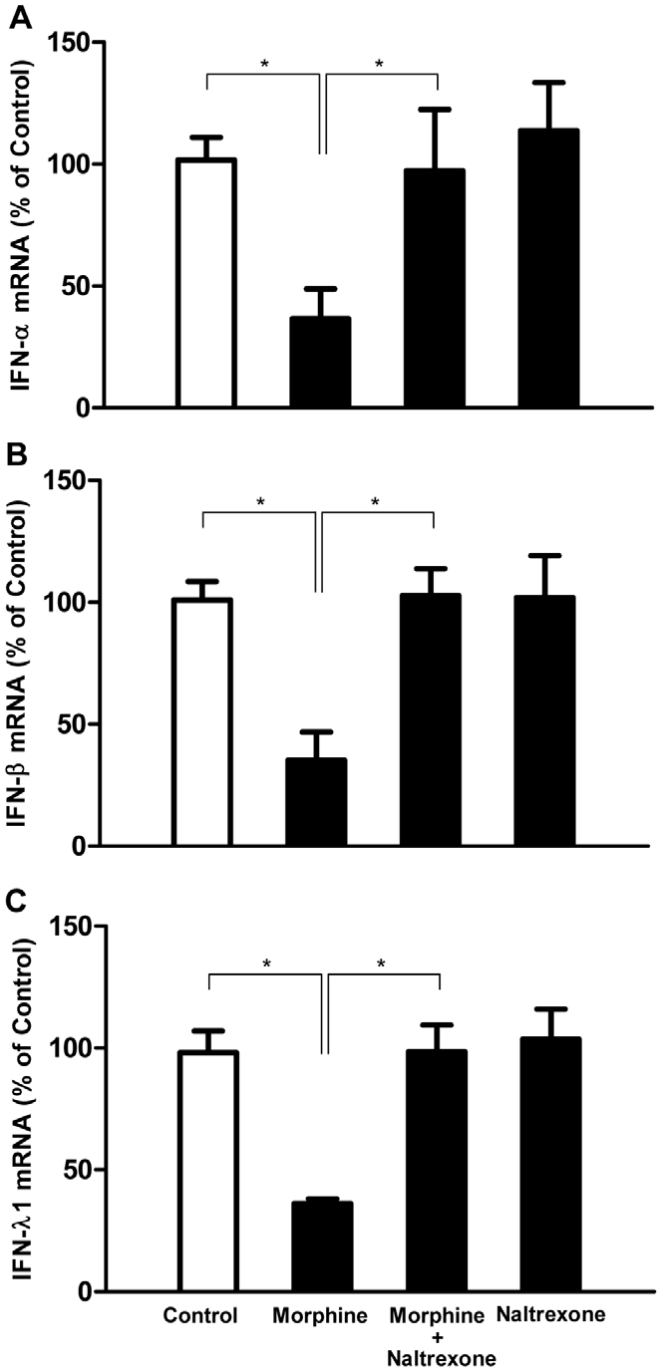
Effect of naltrexone on morphine-mediated down-regulation of IFNs expression. Seven-day-cultured macrophages were incubated with or without naltrexone (10^−8^ M) for 1 h before treatment with or without morphine (10^−10^ M) for 3 h. Cellular RNA was subject to the real-time RT-PCR for IFN-α (A), IFN-β (B) and IFN-λ1 mRNA (C). Data are expressed as mRNA levels in morphine treated cells (percentage of control) those untreated cells and morphine treated cells. The results represent the mean ± SD of three independent experiments. Statistical analysis was performed using one-way analysis of variance, and significance is shown with *P<0.05 (morphine vs control or morphine vs morphine + naltrexone).

IFNs play a key role in host cell innate immunity against viral infections, including HIV. We then examined whether morphine has the ability to inhibit intracellular IFN gene expression in macrophages. Morphine treatment significantly suppressed IFN-α (Fig. 4A), IFN-β (Fig. 4B) and IFN-λ (Fig. 4C) expression in macrophages. These negative effects of morphine on IFNs could be abrogated by naltrexone treatment of macrophages (Fig. 4). Naltrexone alone had little effect on the IFN expression (Fig. 4).

### Morphine suppresses RIG-I, IFN regulatory factors and APOBEC3

**Figure 5:**
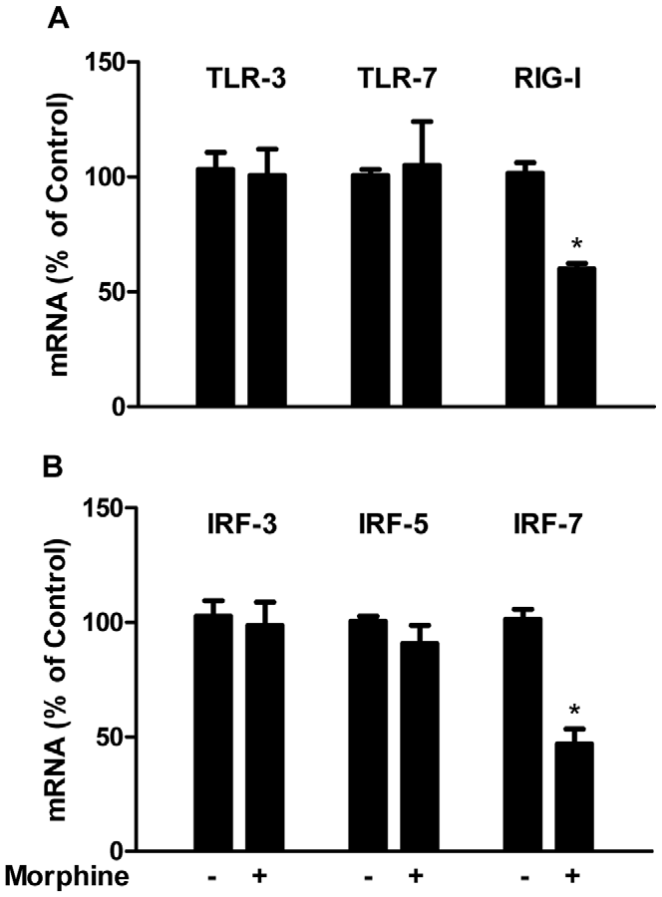
Effect of morphine on TLRs, RIG-I (A) and IRFs (B) expression. Seven-day-cultured macrophages were treated with or without morphine (10^−10^ M) for 3 h, and then cellular RNA was subjected to the real-time RT-PCR for mRNA detection. Data are expressed as mRNA levels in morphine-treated cells (percentage of control) to those in untreated cells. The results represent the mean ± SD of three independent experiments. Statistical analysis was performed by Student's t-test and significance is shown with *P<0.05 (morphine vs control).

**Figure 6:**
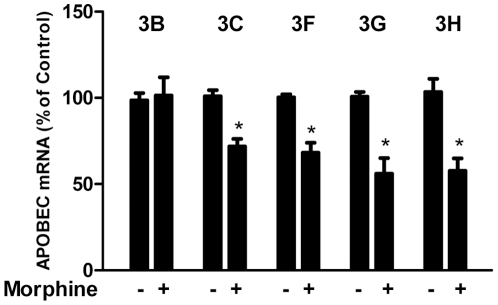
Effect of morphine on APOBEC3B/C/F/G/H mRNA expression. Seven-day-cultured macrophages were treated with or without morphine (10^−10^ M) for 3 h, and then cellular RNA was subjected to the real-time RT-PCR for mRNA detection. Data are expressed as mRNA levels in morphine-treated cells (percentage of control) to those in untreated cells. The results represent the mean ± SD of three independent experiments. Statistical analysis was performed by Student's t-test and significance is shown with *P<0.05 (morphine versus control).

Since TLR and RIG-I play the key roles in IFN-mediated innate immunity against viral infections, we examined whether morphine treatment has the ability to modulate TLR or RIG-I expression. Morphine treatment of macrophages had little effect on TLR-3 or TLR-7 expression (Fig. 5A). In contrast, morphine inhibited RIG-I expression in macrophages (Fig. 5A). We also examined the effect of morphine on IRF expression in macrophages, as IRFs have a crucial role in the regulation of IFNs [40], [41], [42]. Morphine treatment resulted in a significant decrease of IRF-7 expression in macrophages (Fig. 5B). However, morphine had little effect on the expression of IRF-3 and IRF-5 in macrophages (Fig. 5B). Because some of the apolipoprotein B mRNA-editing enzyme catalytic polypeptide-like 3 (APOBEC3) family members have been shown to inhibit the expression of HIV or SIV [43], [44], we thus examined whether morphine has the ability to inhibit APOBEC3 gene expression in macrophages. Morphine-treated macrophages expressed the lower levels of several members (3C, 3F, 3G and 3H) of APOBEC3 family than untreated macrophages (Fig. 6). Morphine had little effect on APOBEC3B expression (Fig. 6).

### Morphine induces SOCS and PIAS

**Figure 7:**
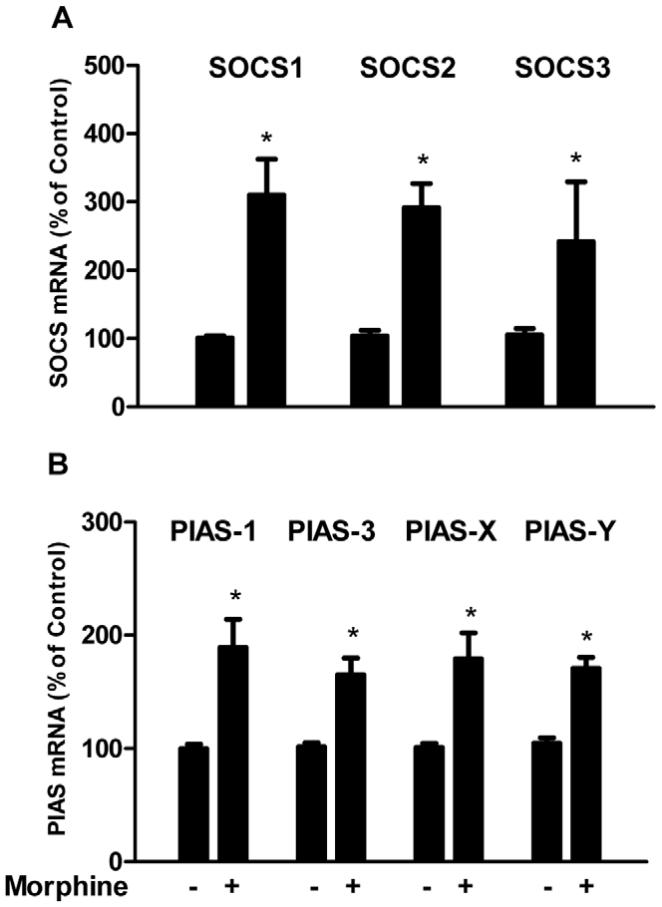
Effects of morphine on SOCS-1, 2, 3 (A), PIAS-1, 3, X and Y (B) expression. Seven-day-cultured macrophages were treated with or without morphine (10^−10^ M) for 3 h, and cellular RNA was subjected to the real-time RT-PCR for mRNA detection. Data are expressed as mRNA levels in morphine-treated cells (percentage of control) to those in untreated cells. The results represent the mean ± SD of three independent experiments. Statistical analysis was performed by Student's t-test and significance is shown with *P<0.05 (morphine versus control).

To further investigate the mechanism(s) involved in the morphine action on HIV and IFN signaling pathway, we investigated effects of morphine on the negative regulatory factors of IFN pathway. SOCS and PIAS are two major families of negative regulators of signal transduction induced by cytokines [45], [46]. SOCS members form a classical negative feedback loop with key actions involving in inhibition of the JAK-STAT signaling cascade, while PIASs are specific inhibitors of STAT signaling. As demonstrated in Figure 7, morphine treatment induced the expression of SOCS-1, 2, 3 and PIAS1, 3, X, Y in macrophages.

## Discussion

Given immunomodulation and immunocompromising effect of opiates, abuse of opiates has been suggested as a cofactor in promoting HIV disease progression. However, much remain to be learned about the mechanisms of opiate-mediated broad influence on host immunity related to control of viral replication. In this study, we showed that morphine significantly inhibited endogenous type I (IFN-α/β) and III (IFN-λ1) IFN expression (Fig. 4), which was associated with increased susceptibility of macrophages to HIV and SIV infection and enhanced virus replication. This morphine effect is specific through the opioid receptor, as the suppression of IFN expression by morphine could be abrogated by naltrexone (Fig. 4). These findings support the earlier reports showing that morphine suppresses Sendai virus-induced IFN-α production by peripheral blood mononuclear cells and monocytes [47], [48]. Our earlier study also showed that morphine inhibited endogenous IFN-α expression and enhanced complete hepatitis C virus replication in human hepatocytes [49]. A novel finding of this study is that morphine inhibited IFN-λ expression in macrophages (Fig. 4). IFN-λ has been shown to inhibit replication of a number of viruses, including HIV [26]. Thus, the finding that morphine inhibited endogenous IFN-λ expression in macrophages provides a sound mechanism for the morphine action on HIV or SIV.

In order to further investigate the mechanism(s) responsible for the action of morphine, we examined the effect of morphine on the expression of TLRs and RIG-I, which recognize viral infections and activate IFN pathway signaling [50]. A recent study showed that purified genomic RNA from HIV induced a RIG-I dependent type I IFN response [51]. Thus, to suppress RIG-I expression by morphine should impair intracellular innate immunity, providing a favorable environment for viral replication. In addition to its negative effect on RIG-I expression, morphine also suppressed the expression of IRF-7, the key regulator of type I IFNs [52]. Similar to type I IFNs, IFN- λ1 is also activated by both IRF-3 and IRF-7 [53]. IRFs not only recognize the elements in the IFN promoter to modulate the expression of type I IFN genes selectively, but also regulate the IFN-stimulated response element (ISRE) in some of IFN-stimulated genes (ISGs), leading to induction of an antiviral state [54], [55]. We were particularly interested in IRF-3 and IRF-7, as IRF-3 and IRF-7 are the key regulators of type I IFN gene expression induced by viruses [56]. IRF-7 is the master regulator of type I IFN-dependent immune response, as it not only induces IFN-α expression, but also activates many antiviral ISGs [52], [57]. Therefore, the suppression of IRF-7 expression in macrophages by morphine treatment explains inhibitory effect of morphine on both type I and type III IFN expression.

APOBEC3 family members are cellular cytidine deaminases that have the ability to inhibit the mobility of HIV [43], [44]. Among the APOBEC3 family members, APOBEC3G, APOBEC3F and APOBEC3H have been identified to have the ability to restrict HIV replication in both CD4^+^ T cells and macrophages [58], [59], [60], [61]. APOBEC3G can either edit the newly synthesized viral DNA or have an inhibitory effect through lethal editing of nascent reverse transcripts of the HIV life cycle [62], [63], [64]. APOBEC3F also encodes an antiretroviral protein that is selectively packaged into HIV virions and profoundly inhibits HIV infectivity [65]. APOBEC3B and APOBEC3C have been shown to act as the potent inhibitors of SIV replication [44]. Thus, the suppression of several key members of APOBEC3 family in macrophages by morphine justifies the enhancing effect of morphine action on HIV or SIV infection and replication.

To further explore the mechanisms involved in morphine-mediated enhancement of AIDS virus infection of macrophages, we attempted to determine whether morphine modulates the expression of the negative regulators of the JAK-STAT signaling pathway. It is known that the JAK-STAT signaling pathway is the major pathway for IFN-mediated signaling and activation of gene expression [66]. IFNs through binding to their specific receptors activate JAK-STAT pathway, which regulates the expression of immune system genes [67]. Morphine treatment not only induced the expression of SOCS-1, SOCS-2 and SOCS-3, but also enhanced the expression of PIAS-1, PIAS-3, PIAS-X and PIAS-Y, the potent suppressors of the JAK-STAT signaling cascade [45], [46], [68]. These findings support our earlier *in vivo* investigation, showing that the heroin users had significantly higher levels of SOCS and PIAS than the control subjects [69].

Taken together, our study provides compelling experimental evidence that morphine enhances AIDS virus replication in macrophages through the modulation of multiple factors in IFN signaling pathway at both cellular and molecular levels. Although additional mechanisms might also be involved in the morphine action on AIDS virus, to suppress the expression of endogenous IFNs and IFN-inducible antiviral genes should account for much of morphine-mediated HIV or SIV enhancement in macrophages. Because morphine exerts a profound and detrimental effects on host cell innate immunity that has a critical role in restricting HIV or SIV replication in macrophages, it is likely that opiate abuse has the ability to alter the course of HIV disease progression.
